# Comparison of health inequalities according to socioeconomic status: findings from the fourth Korean working condition survey (a cross-sectional study)

**DOI:** 10.11604/pamj.2023.44.107.29516

**Published:** 2023-02-27

**Authors:** SangJin Park, Minsu Ock, Ahra Kim, Joo Hyun Sung

**Affiliations:** 1 Department of Occupational and Environmental Medicine, Gyeongsang National University Changwon Hospital, Gyeongsang National University College of Medicine, Jinju, Korea,; 2 Department of Preventive Medicine, Ulsan University Hospital, University of Ulsan College of Medicine, Ulsan, Korea,; 3 Department of Biology, Institute of Health Sciences, Gyeongsang National University College of Medicine, Jinju, Korea

**Keywords:** Lorenz curve, Gini index, socioeconomic status, health inequality

## Abstract

**Introduction:**

this study aims to examine health inequalities among Korean workers by sex, age, education, monthly income, occupation, and employment type and identify groups of workers who may be neglected in the process of resolving health inequalities.

**Methods:**

we used data from the Fourth Korean Working Condition Survey conducted by the Korea Occupational Safety and Health Research Institute and compared the number of health symptoms among various groups using the t-test and one-way analysis of variance to determine their health status. We also calculated the Gini index of the number of health symptoms of each group and plotted the Lorenz curve to illustrate health inequalities.

**Results:**

we found that the number of health symptoms was higher in groups with lower socioeconomic status (e.g., female, blue-collar workers, older, low education, low monthly income, and self-employed workers). However, the Gini index and Lorenz curve with respect to socioeconomic status indicated that health inequalities were higher among white-collar and permanent workers vis-à-vis blue-collar and self-employed workers, respectively. Further, it was found that health inequalities were higher among males than females with respect to the same occupational groups and employment types.

**Conclusion:**

general health policies are often targeted at the socially and economically vulnerable group, but according to the results of this study, it can be considered that there may be subjects who are vulnerable to health problems even in groups that are not socioeconomically vulnerable.

## Introduction

Health inequality is defined as “differences in health status or in the distribution of health resources between different population groups, arising from the social conditions in which people are born, grow, live, work and age” [1]. In 1946, the World Health Organization (WHO) constitution enshrined that “the enjoyment of the highest attainable standard of health is one of the fundamental rights of every human being without distinction of race, religion, political belief, economic or social condition” [2]. However, with the development of societies, health inequalities continue to emerge.

An evaluation report on environmental health inequalities in Europe by the WHO Regional Office assessed health inequalities with respect to sex, age, income, education, occupation, and social status [3] and found that health inequalities from exposure to noise and second-hand smoke were related to income levels and injury mortality rates of males were three times higher than that of females. Developed countries have established a variety of policies to alleviate health inequalities. In the United States, a health protection strategy called “healthy people 2020” was established to alleviate health inequalities [4]. In the United Kingdom, one of the ten core principles of the National Health Service emphasizes the need to reduce health inequalities [5]. Several studies have been conducted in Korea in response to an increased perception of health inequality in the country [6,7]. However, most of these studies are biased towards identifying health inequalities among different social classes and their results are limited to identifying health vulnerabilities facing blue-collar workers or low socioeconomic status groups.

Therefore, this study aims to examine health inequalities among Korean workers with respect to sex, age, education, monthly income, occupation, and employment type using the Fourth Korean Working Conditions Survey (KWCS) data and identify groups of workers who may be neglected in the process of resolving health inequalities.

## Methods

**Study design:** this study was conducted as a cross-sectional study using the fourth KWCS data. The purpose of the study was to investigate health inequalities among Korean workers with respect to sex, age, education, monthly income, occupation, and employment type, and to identify groups of workers who may be neglected in the process of resolving health inequalities.

**Study setting and population:** this study used data from the fourth KWCS, which was conducted by the Korea OSHRI from June to September 2014. The Korea OSHRI is located in the South Korea in East Asia. In 2006, the first KWCS was conducted based on the European working condition survey and has been conducted four times as of 2014. The validity and reliability of the KWCS have been verified in a past study [8]. The survey was conducted by professional researchers trained in interviewing methods. The interviewing methods used for the fourth KWCS were the paper and pen interview (PAPI) and computer assisted personal interview (CAPI) methods. Across 17 cities, 50007 subjects participate in the study, and they were interviewed by the researchers over a four-month period. Korean working condition survey targets all employees aged 15 or older in Korea. Based on the 2010 population and housing census data and the list of new apartments, samples were extracted after stratifying the subjects. In this study, since the analysis was performed on adults, subjects under the age of 20 were excluded. In addition, since soldiers are included from officers to private soldiers, the classification of occupations is ambiguous, so they were excluded. Finally, we excluded 9955 subjects with missing values (sex, age, education, monthly income, occupation, and employment type, aged under 20, soldier), leaving 40052 individuals (21193 men, 18859 women).

**Variables and data resource:** to assess the general characteristics, we used the data related to sex, age, education, monthly income, occupation, and employment type from the fourth KWCS. Then, we used 13 health symptom questionnaires from the fourth KWCS to assess health inequality.

**General characteristics:** to assess inter- and intra-group health inequality, we classified all the variables into categorical variables. Age was divided into “20-29.9,” “30-39.9,” “40-49.9,” “50-59.9,” and “60 and above”; education level into “middle school graduate and below,” “high school graduate,” and “college and above”; monthly income (in USD) into “below 1000,” “1000-1999,” “2000-2999,” “3000-3999,” “4000 and above,” and “no answer or unknown.” Occupation was divided into “blue-collar workers” and “white-collar workers.” Here, blue-collar workers include agricultural workers, fishery workers, operators, assembly workers, and simple laborers (e.g., guard, sweeper, deliveryman, and driver) and white-collar workers include managers, experts, office workers, engineers, service workers, and sales workers. Finally, employment type was divided into “self-employed,” “temporary worker,” and “permanent worker.”

**Socioeconomic status:** to assess health inequality with respect to socioeconomic status (SES), we reclassified the subjects into three groups (low, intermediate, and high SES groups). We used age, education, and monthly income to determine the SES. First, we analyzed the frequency of number of health symptoms by age, education, and monthly income. Then, we divided age, education, monthly income into “good SES” and “poor SES” according to the median value of the number of subjects for each variable. “Less than 50 years of age,” “above high school education,” and “more than 2000 USD monthly income” were classified as good SES, while others were classified as poor SES. Next, we classified the subjects satisfying all the above three conditions of poor SES into the “low SES” group, those satisfying all three conditions of good SES into the “high SES” group, and the remainder into the “intermediate SES” group.

**Health symptom questionnaire:** in the KWCS, if a subject answered, “yes” to the question, “During the past 12 months, have you had any of the following health problems?” they were considered to have health problems. The health problems were divided into 13 categories: i) hearing function problem; ii) dermatologic problem; iii) lower back pain; iv) muscular pain in upper extremities such as shoulder, neck, and arm; v) muscular pain in lower extremities such as hip, leg, knee, and foot; vi) headache or eye strain; vii) abdominal pain; viii) dyspnea; ix) cardiovascular disease; x) trauma or accident; (xi) depression or anxiety disorder; xii) whole-body fatigue; xiii) insomnia or sleep disorder.

**Figure 1:**
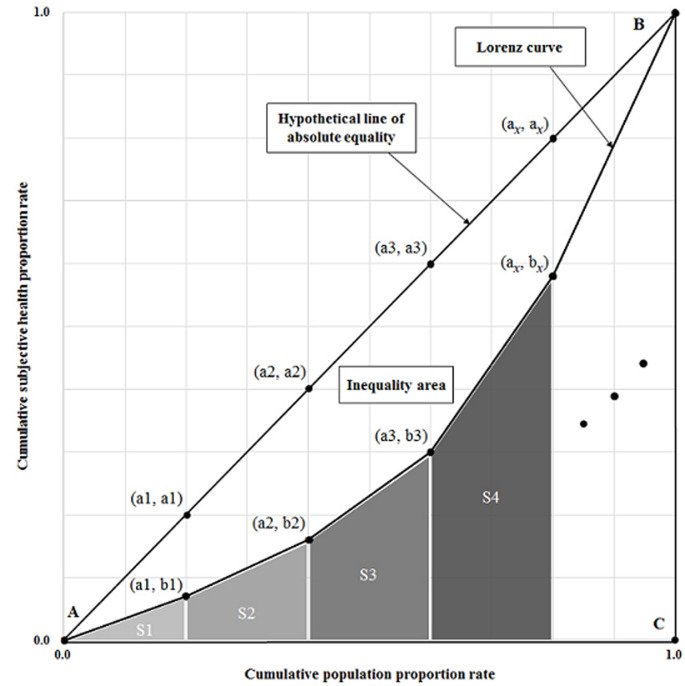
Lorenz curve and inequality area

**Data analysis:** we compared the number of symptoms based on sex and occupation using the student´s t-test. We also compared the number of symptoms based on age, education, monthly income, and employment type using the one-way ANOVA test. The p-value of lesser than 0.05 was considered as statistically significant. To assess health inequality, we calculated the Gini index of the number of health symptoms. We also plotted a Lorenz curve to visually identify health inequality. The Gini index [9,10] and the Lorenz curve [11] are widely used indicators of income inequality in economics. In recent studies, these indicators have been used to assess inequality in other fields [12,13]. This study used these indicators to determine the degree of health inequality. We used International business machines statistical package for the social sciences (IBM SPSS) statistics for Windows, version 24.0 (IBM, SPSS Inc., Armonk, NY, USA) to conduct the student´s t-test and the ANOVA test. We also used Excel 2016 (Microsoft, Redmond, WA, USA) to calculate the Gini index and to draw the Lorenz curve. We first drew the Lorenz curve and then calculated the Gini index. To draw the Lorenz curve, we set the x-axis as “cumulative population proportion rate” and the y-axis as “cumulative subjective health proportion rate.” To obtain “cumulative population proportion rate,” we calculated the total number of subjects per number of symptoms. To obtain “cumulative subjective health proportion rate,” we transformed the subjective health symptom range from “0-13” to “0.0-1.0.” The Gini index [14] measures the area between the Lorenz curve and the hypothetical line of absolute equality (triangle ABC in Figure 1), expressed as a percentage of the maximum area under the line (Figure 1). It is expressed as a value lying within a range of 0-1, such that the closer the value is to “0,” the higher the degree of equality, and the closer it is to “1,” the higher the degree of inequality. The formula for calculating the Gini index is as follows: Gini index = inequality area/triangular area; - inequality area: the area between a hypothetical line of absolute equality and the Lorenz curve; - triangular area: the area of a triangle below a hypothetical line of absolute equality. In this study, as the cumulative subjective health proportion rate was not a continuous variable, we did not use an integral function to measure the inequality area. Instead, we used the following simple numerical area calculation method in Figure 1. Gini index = (area of triangle ABC - area under Lorenz curve)/area of triangle ABC; Gini index = 1 - area under Lorenz curve/area of triangle ABC; Gini index = 1 - area under Lorenz curve/0.5; Gini index = 1 - 2 x area under Lorenz curve; Gini index = 1 - 2 x [(1/2 x a1 x b1) (S1) + {1/2 x (b1 + b2) x (a2 - a1)} (S2) + {1/2 x (b2 + b3) x (a3 - a2)} (S3) + ……]; Gini index = 1- [(a1 x b1) + {(b1 + b2) x (a2 - a1)} + {(b2 + b3) x (a3 - a2)} + ……].


$$\text{Gini index}=1-\left(a1\times b1\right)-\sum_{1}^{13}\left\{\left(b_{x}+b_{x+1}\right)\times \left(a_{x+1}-a_{x}\right)\right\}$$

## Results

**Frequency of number of health symptoms in each category based on the different population:** the number of health symptoms was higher among females and blue-collar workers than males (p<0.001) and white-collar workers (p<0.001), respectively. Moreover, the number of health symptoms increased with increase in age (p<0.001) and decrease in educational level (p<0.001) and monthly income (p<0.001). According to employment type, the number of health symptoms was highest among the self-employed, followed by temporary and permanent workers (p<0.001) (Table 1).

**Table 1:** frequency of number of health symptoms with respect to sex, age, education, monthly income, occupation, and employment type

Characteristics	Respondents (n=40052)	Mean	Standard deviation	P-value
**Sex**				
Male	21193 (52.9)	1.33	1.65	< 0.001
Female	18859 (47.1)	1.59	1.74	
**Age**				
20 - 29.9	3688 (9.2)	0.80	1.32	< 0.001
30 - 39.9	7923 (19.8)	1.00	1.43	
40 - 49.9	10959 (27.4)	1.34	1.65	
50 - 59.9	9441 (23.6)	1.57	1.66	
60 or above	8041 (20.0)	2.22	1.87	
**Education**				
Middle school graduate or below	7542 (18.8)	2.37	1.84	< 0.001
High school graduate	16021 (40.0)	1.46	1.67	
College or above	16489 (41.2)	1.03	1.46	
**Monthly income (USD)**				
Under 1000	8254 (20.6)	1.97	1.90	< 0.001
1000 - 1999	15038 (37.5)	1.42	1.66	
2000 - 2999	9720 (24.3)	1.24	1.56	
3000 - 3999	4511 (11.3)	1.22	1.57	
4000 or above	2529 (6.3)	1.21	1.56	
**Occupation**				
Blue-collar worker*	13769 (34.4)	1.95	1.85	< 0.001
White-collar worker†	26283 (65.6)	1.19	1.54	
**Employment type**				
Self-employed worker	14314 (35.7)	1.79	1.80	< 0.001
Temporary worker	6094 (15.2)	1.56	1.75	
Permanent worker	19644 (49.1)	1.18	1.54	

* Blue-collar workers include agricultural workers, fishery workers, operators, assembly workers, and simple laborers (e.g., guard, sweeper, deliveryman, and driver); †White-collar workers include managers, experts, office workers, engineers, service workers, and sales workers

**Gini index based on the different population:** the Gini index values were higher for males and white-collar workers vis-à-vis females and blue-collar workers, respectively. They decreased with increase in age and decrease in educational level, monthly income, and socioeconomic status. According to employment type, the Gini index value was highest among permanent workers, followed by temporary and self-employed workers (Table 2).

**Table 2:** Gini index with respect to sex, age, education, monthly income, occupation, employment type, and socioeconomic status

Characteristics	Gini index of low socioeconomic status	Gini index of intermediate socioeconomic status	Gini index of high socioeconomic status
**Male**			
Occupation			
Blue-collar worker*	0.728	0.804	0.829
White-collar worker†	0.813	0.890	0.895
Employment type			
Self-employed worker	0.717	0.831	0.865
Temporary worker	0.773	0.859	0.852
Permanent worker	0.811	0.867	0.892
**Female**			
Occupation			
Blue-collar worker*	0.670	0.798	0.810
White-collar worker†	0.763	0.848	0.859
Employment type			
Self-employed worker	0.689	0.806	0.831
Temporary worker	0.721	0.853	0.855
Permanent worker	0.756	0.856	0.867

* Blue-collar workers include agricultural workers, fishery workers, operators, assembly workers, and simple laborers (e.g. guard, sweeper, deliveryman, and driver); †White-collar workers include managers, experts, office workers, engineers, service workers, and sales workers

**Gini index, and lorenz curve based on the different population:** according to occupation, both male and female blue-collar and white-collar workers tended to have a larger Gini index as their socioeconomic status increased. The Gini indexes for all the cases were greater for males and white-collar workers than females and blue-collar workers, but the difference between the indexes of intermediate and high SES was not large. According to employment type, both male and female self-employed and permanent workers tended to have a larger Gini index as their socioeconomic status increased. In the case of male temporary workers, the Gini index was higher for intermediate SES than high SES, but the difference was not large. Temporary and high SES workers had a smaller Gini index for male vis-à-vis female. However, in all other cases the indexes were larger for males than females. Further, in all the cases, the Gini index was increasing in order of self-employed workers, temporary, and permanent workers (Table 3).

**Table 3:** Gini index of occupation and employment type with respect to sex and socioeconomic status

Characteristics	Gini index of low socioeconomic status	Gini index of intermediate socioeconomic status	Gini index of high socioeconomic status
**Male**			
Occupation			
Blue-collar worker*	0.728	0.804	0.829
White-collar worker†	0.813	0.890	0.895
Employment type			

* Blue-collar workers include agricultural workers, fishery workers, operators, assembly workers, and simple laborers (e.g. guard, sweeper, deliveryman, and driver); †White-collar workers include managers, experts, office workers, engineers, service workers, and sales workers

**Main results:** the Health problems may appear differently according to socioeconomic factors such as sex, age, education, monthly income, occupation, employment type (p<0.001). The lower socioeconomic status group have more health problems. However, the higher socioeconomic status group may have more health inequality.

## Discussion

Health inequality is an easily encountered problem in modern society. In this situation, examining the differences in health levels among groups of workers can help determine the current status and cause of health inequality, and thus provide solutions for the problem [7]. In this study, we analyzed work-related health symptoms to examine health level differences among Korean workers and found health inequalities in various groups using the Gini index and the Lorenz curve. Previous studies have analyzed and offered solutions to health problems existing in the lower socioeconomic strata based on factors such as education level and income level [3,4,6,15-19]. The results of this study confirm that people of lower socioeconomic status live relatively less healthy lives. However, in this study, we further divided each factor into several categories and evaluated health inequality in each category. We found that inequality was highest among male white-collar workers with respect to occupation and male permanent workers with respect to employment type. This is in contrast to the lowest levels of health inequalities found among female blue-collar workers with respect to occupation and female self-employed workers with respect to employment type, although these groups are known to be relatively vulnerable to health equity. In addition, health inequalities within groups tended to be larger with higher SES. These results are likely due to the characteristics of the target population groups. In low SES groups, there is not much deviation in education and monthly income. On the other hand, in high SES group, the deviation in education and monthly income is very large. In education, for example, the “college or above” group includes colleges to graduate school, where the qualifications of subjects range from associate degrees to doctors. The work performed by each member is expected to vary widely as well. As regards monthly income, the “4000 or above” group is also expected to have a large deviation as it includes all subjects earning above 4000 USD. Therefore, it can be inferred that the difference between the lowest and highest level of workers in the high SES group may be much larger than the difference among the members in the low SES group, thereby increasing the level of health inequalities in the high SES group accordingly. In the stratified analysis with respect to sex, occupation, and employment type, there was a tendency for health inequalities to increase consistently with higher SES, which supports our opinion.

As a result, the workers who experience health inequalities in high SES groups is likely to be marginalized by existing research and policies that are usually aimed at workers in low SES groups. In fact, research on musculoskeletal disorders, such as visual display terminal syndrome (VDT syndrome), among white-collar workers has been conducted only recently [20-23]. Moreover, problems such as mental stress among white-collar workers have just been brought to light [24,25]. This suggests the possibility that some workers are still marginalized. Therefore, there may be reverse discriminations in policymaking. Few studies have analyzed health inequalities with respect to socioeconomic strata by classifying the subjects into further categories. This study shows that there is a possibility that some subjects who experience health inequality despite their higher socioeconomic status are likely to be marginalized in the process of resolving health inequalities. Further research is needed to identify the factors that should be preferentially considered and managed to overcome health inequalities within specific population groups. Although the results of this study were significant, there are some limitations. First, as the KWCS data is on Korean workers, this study cannot be applied to other countries in the same way. In particular, health inequalities may vary not only across socioeconomic dimensions, but also across other dimensions like political and religious identity. Therefore, it is difficult to generalize and apply these findings to other countries [26,27]. Third, in a validity survey conducted in the past for KWCS, it was found that the response rate was lower than that of EWCS. Therefore, this study also has a possibility of lowering the reliability compared to the EWCS analysis. Second, as this study is a cross-sectional in nature, causal relationships cannot be explained. Finally, as we used only the KWCS data in this study, the factors not included in it cannot be considered. However, this study has the advantage of being highly reliable as it uses data based on a sample representative of Korean workers. Further, this study makes a novel attempt to visualize and quantitatively compare health inequalities using the Lorenz curve and Gini index. Moreover, in contrast to previous studies that have identified health inequalities among different socioeconomic strata, this study found that health inequalities could exist within the same strata.

## Conclusion

In line with the findings of past studies, we found that low-SES workers live relatively less healthy lives. Analyzing further by sex, occupation (highest inequality among male white-collar workers), and employment type (highest inequality among male permanent workers), we found that some high-SES workers may suffer from health inequalities. Furthermore, health inequalities within groups tended to be larger with higher SES. Most countries are proposing policies to alleviate health inequalities through institutional methods. Nevertheless, these policies target only low-SES workers, and, as shown here, some high-SES workers may be marginalized. Our study can provide data for developing methods to include such workers in the ambit of research and policies.

### What is known about this topic

- *Health problems may appear differently according to socioeconomic factors (such as sex, age, education, monthly income, occupation, employment type);*
- *The lower socioeconomic status group have more health problems*.

### What this study adds

- *The higher socioeconomic status group may have more health inequality;*
- *New methods such as Gini index, Lorenz curve can be used to assess health inequality*.
